# Supplementary figures and images for: Inhibition of experimental autoimmune uveitis by intravitreal AAV-Equine-IL10 gene therapy
Source: PLoS One. 2022 Aug 18;17(8):e0270972. doi: 10.1371/journal.pone.0270972 (PMC9387812; doi:10.1371/journal.pone.0270972)

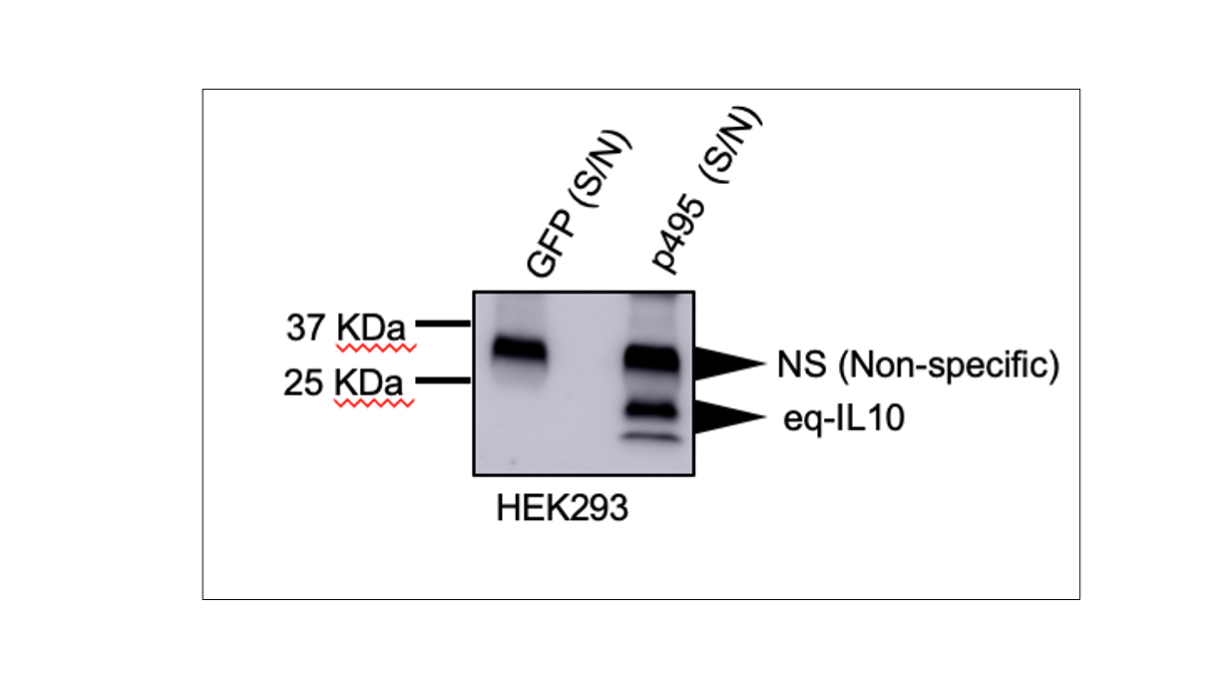

Supplement: S1 Fig — A. A Western blot was used to detect Equine-IL10 protein following transfection of human embryonic kidney 293 cells (HEK293). Equine-IL10 protein (Equine-IL10) was detected in the supernatant of cultured HEK293 cells. KDa (kilodaltons). GFP (green fluorescent protein). p459 (Equine IL-10 plasmid). B. Uncropped raw image of Equine IL10 Western blot. (ZIP) [file pone.0270972.s001.zip › S1a_Fig.tiff]

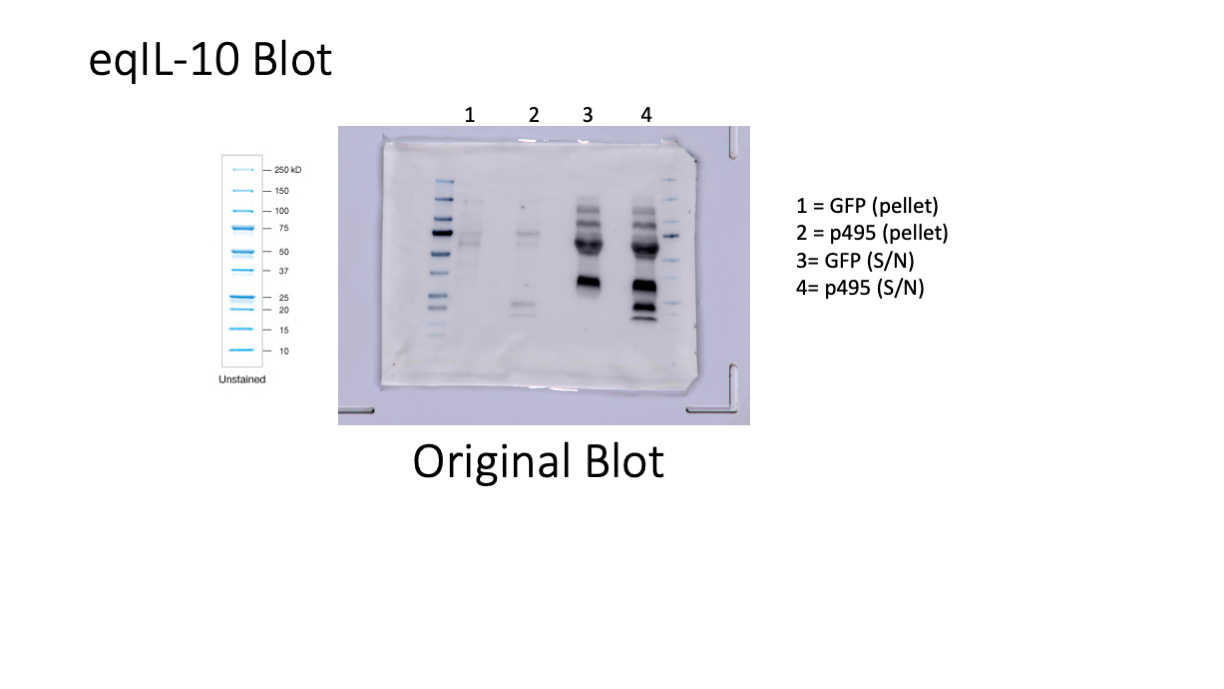

Supplement: S1 Fig — A. A Western blot was used to detect Equine-IL10 protein following transfection of human embryonic kidney 293 cells (HEK293). Equine-IL10 protein (Equine-IL10) was detected in the supernatant of cultured HEK293 cells. KDa (kilodaltons). GFP (green fluorescent protein). p459 (Equine IL-10 plasmid). B. Uncropped raw image of Equine IL10 Western blot. (ZIP) [file pone.0270972.s001.zip › S1b_Fig.tiff]

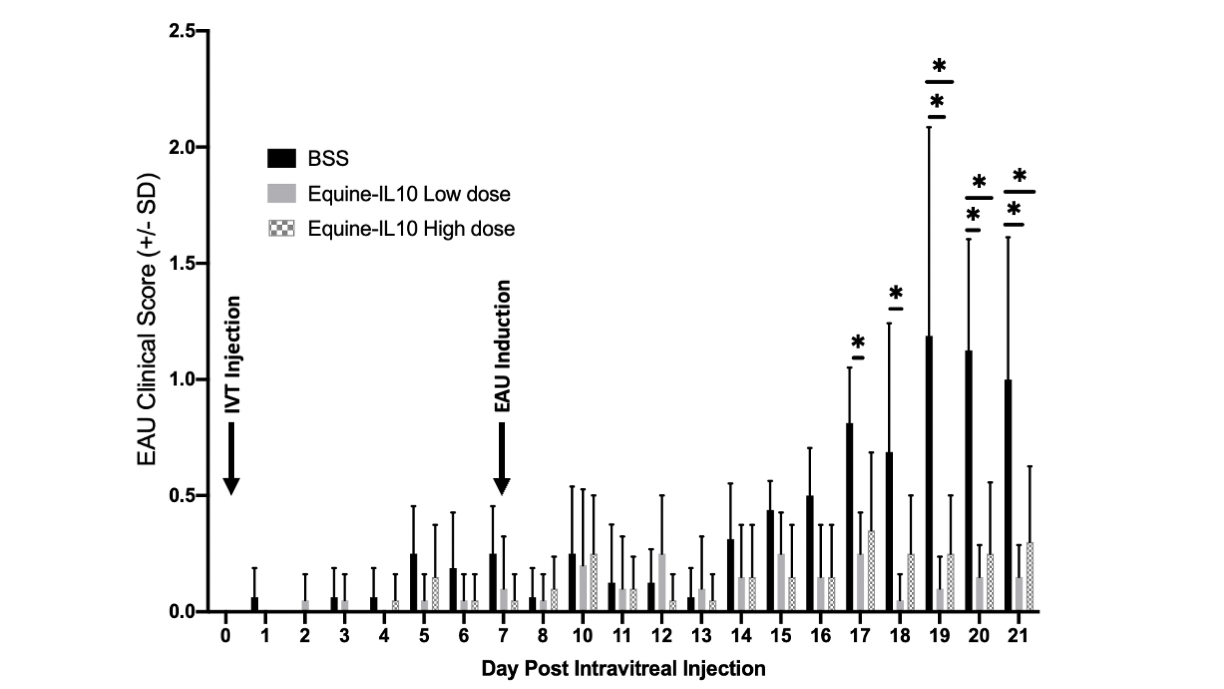

Supplement: S2 Fig — Clinical examination inflammatory scores for daily examination after intravitreal injection. EAU was induced in all rats on 7 days after the intravitreal injections. Peak inflammation was seen on days 17–21 after intravitreal injections (10–14 days post EAU induction); * p < 0.05, Pairwise Wilcoxon tests. (TIFF) [file pone.0270972.s002.tiff]

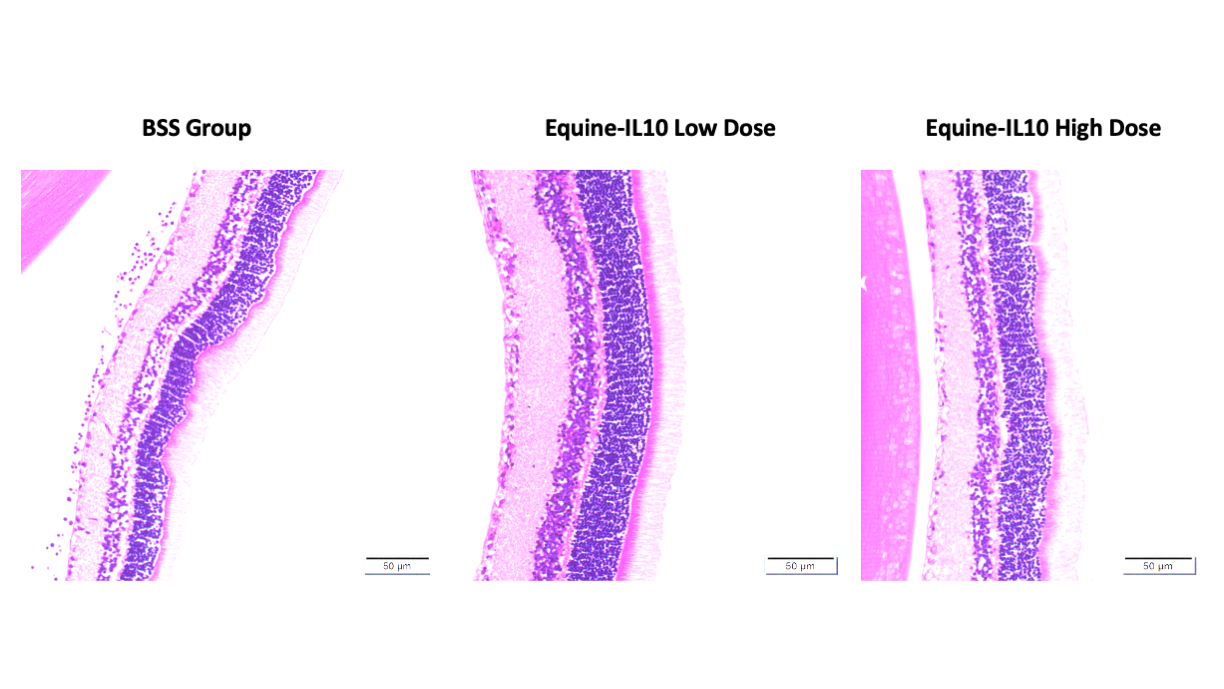

Supplement: S3 Fig — Representative images of ocular histology demonstrate inflammatory cell infiltration vitreous body, and retina in experimental autoimmune uveitis (EAU) BSS eyes, with almost infiltration of inflammatory cells observed high dose and low dose Equine-IL10 treated EAU eyes. (hematoxylin & eosin staining, original magnification: 20x). (TIFF) [file pone.0270972.s003.tiff]

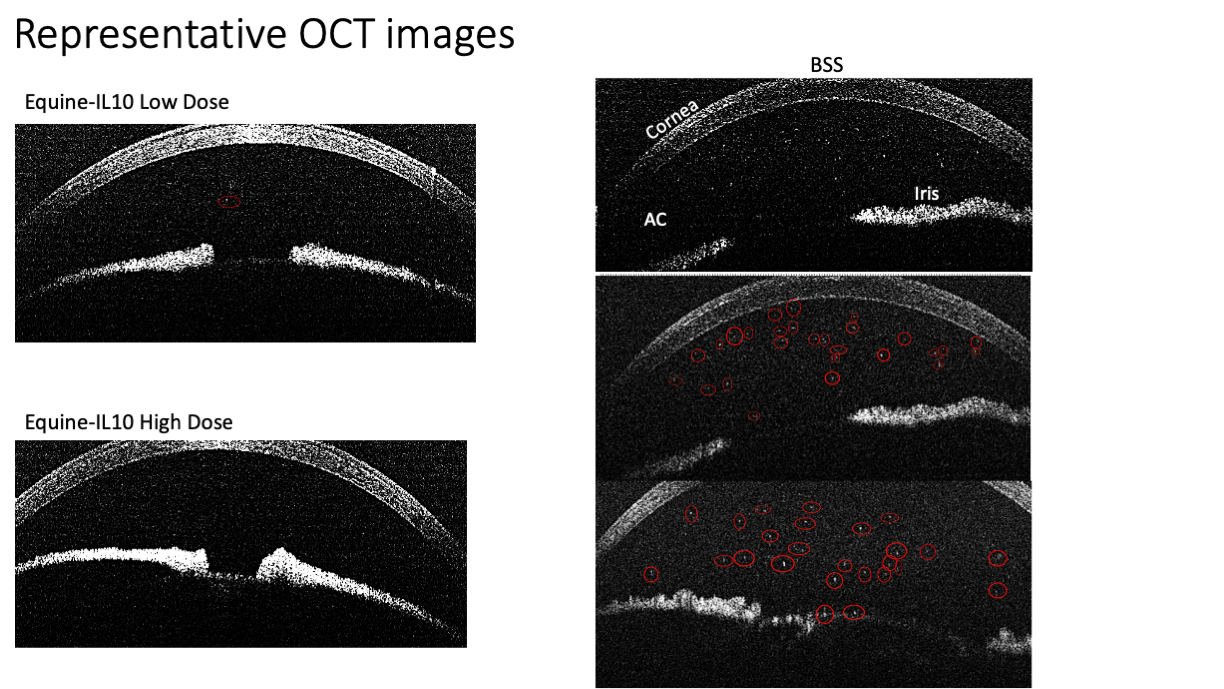

Supplement: S4 Fig — The red circles used to demonstrate cells in the anterior chamber. The iris, cornea and anterior chamber (AC) are labeled in the top right image. (TIFF) [file pone.0270972.s004.tiff]
