# Supplementary material for: Inhibition of experimental autoimmune uveitis by intravitreal AAV-Equine-IL10 gene therapy
Source: PLoS One. 2022 Aug 18;17(8):e0270972. doi: 10.1371/journal.pone.0270972 (PMC9387812; doi:10.1371/journal.pone.0270972)
Supplement: Attachment — Submitted filename: Response to Reviewers eqIL10 Final.docx [file pone.0270972.s005.docx]

**Response to Editor, Manuscript: PONE-D-21-37491**

1. Please ensure that your manuscript meets PLOS ONE's style requirements, including those for file naming. The PLOS ONE style templates can be found at

<https://journals.plos.org/plosone/s/file?id=wjVg/PLOSOne_formatting_sample_main_body.pdf>  and

<https://journals.plos.org/plosone/s/file?id=ba62/PLOSOne_formatting_sample_title_authors_affiliations.pdf>

*Response: We reviewed the manuscript and made changes to meet PLOS ONE’s style requirements.*

2. We note that the grant information you provided in the ‘Funding Information’ and ‘Financial Disclosure’ sections do not match.

When you resubmit, please ensure that you provide the correct grant numbers for the awards you received for your study in the ‘Funding Information’ section.

*Response: We reviewed and corrected the grant information in the ‘Funding Information’ and ‘Financial Disclosure’ sections.*

3. PLOS ONE now requires that authors provide the original uncropped and unadjusted images underlying all blot or gel results reported in a submission’s figures or Supporting Information files. This policy and the journal’s other requirements for blot/gel reporting and figure preparation are described in detail at <https://journals.plos.org/plosone/s/figures#loc-blot-and-gel-reporting-requirements> and <https://journals.plos.org/plosone/s/figures#loc-preparing-figures-from-image-files>. When you submit your revised manuscript, please ensure that your figures adhere fully to these guidelines and provide the original underlying images for all blot or gel data reported in your submission. See the following link for instructions on providing the original image data: <https://journals.plos.org/plosone/s/figures#loc-original-images-for-blots-and-gels>.

In your cover letter, please note whether your blot/gel image data are in Supporting Information or posted at a public data repository, provide the repository URL if relevant, and provide specific details as to which raw blot/gel images, if any, are not available. Email us at [plosone@plos.org](mailto:plosone@plos.org) if you have any questions.

*Response: All supporting data and uncropped and unadjusted blot/gel images have been uploaded in the Dryad database: Gilger, Brian (2022), AAV-eqIL-10 in EAU, Dryad, Dataset,* [*https://doi.org/10.5061/dryad.q83bk3jkq*](https://doi.org/10.5061/dryad.q83bk3jkq)

4. In your Data Availability statement, you have not specified where the minimal data set underlying the results described in your manuscript can be found. PLOS defines a study's minimal data set as the underlying data used to reach the conclusions drawn in the manuscript and any additional data required to replicate the reported study findings in their entirety. All PLOS journals require that the minimal data set be made fully available. For more information about our data policy, please see <http://journals.plos.org/plosone/s/data-availability>.

Upon re-submitting your revised manuscript, please upload your study’s minimal underlying data set as either Supporting Information files or to a stable, public repository and include the relevant URLs, DOIs, or accession numbers within your revised cover letter. For a list of acceptable repositories, please see <http://journals.plos.org/plosone/s/data-availability#loc-recommended-repositories>. Any potentially identifying patient information must be fully anonymized.

*Response: All supporting data and uncropped and unadjusted images have been uploaded in the Dryad database: Gilger, Brian (2022), AAV-eqIL-10 in EAU, Dryad, Dataset,* [*https://doi.org/10.5061/dryad.q83bk3jkq*](https://doi.org/10.5061/dryad.q83bk3jkq)*. Please use this link during peer review:* [*https://datadryad.org/stash/share/WroYYOMKjFp66-RS_w5bspj9dlFDDktOux5BQkAHOPQ*](https://datadryad.org/stash/share/WroYYOMKjFp66-RS_w5bspj9dlFDDktOux5BQkAHOPQ)*.*

We will update your Data Availability statement to reflect the information you provide in your cover letter.

5. Please upload a new copy of Figures 4 as the detail is not clear. Please follow the link for more information: <https://blogs.plos.org/plos/2019/06/looking-good-tips-for-creating-your-plos-figures-graphics/>" <https://blogs.plos.org/plos/2019/06/looking-good-tips-for-creating-your-plos-figures-graphics>.

*New Figure 4 was composed and uploaded*

**Response to Reviewers, Manuscript: PONE-D-21-37491**

**Reviewer #1:** The authors address the significant unmet need for an effective, local, non-corticosteroid treatment option for non-infection uveitis. The goal of the study is to use a rat model of uveitis to study feasibility of their gene therapy approach prior to use in equine recurrent uveitis (ERU). The authors test the ability of two doses of an AAV8 vector expressing IL-10 to prevent EAU in Lewis rats. They conclude that intravitreal injection of both low and high dose vectors significantly decreased ocular inflammation and retinal damage. The manuscript is well written. The intro and discussion are thorough, and figures are appropriate. There are a few experimental design concerns that limit the significance of the conclusions. Specifically, that the treatment effect is local, and that the treatment approach is sufficient to treat uveitis in the presence of an established anti-retinal T cell population. Despite these concerns, this is an interesting study.

*Response: Thank you for your comments*

Experimental design:
1. Immunosuppression administered prior to or during the immunization phase of EAU can prevent development of the anti-ocular T cell population. In this study, it would be important to show that the local treatment approach (administered prior to uveitis induction) did not prevent the development of the expected autoreactive T cells. Ultimately for this gene therapy approach to be effective it must function in the context of the anti-ocular effector T cell population. Currently that data could also be interpreted to mean that the treatment prevented the generation of the anti-ocular T cell population at the time of immunization. Optional experiments would be to treat rats with IVT AAV8-IL-10 and then use adoptive transfer to generate EAU. Alternatively, T cell ELISPOT from immunized rats could be used to demonstrate the autoreactive T cells were present.

*Response: These are excellent comments and we added discussion points around this. Although unproven, our hypothesis is that the intravitreal IL-10 gene transfer and expression is limited primarily to the eye. This is supported by our published work using AAV8 in an EAU rat model in which we failed to detect viral genomes in the liver, kidney, brain, spleen and lymph nodes even when using 30X more virus than the low dose herein (PMID: 31882729). In fact, despite the sensitivity of PCR, the detection levels of viral genomes in the eyes injected with low dose herein, while trending higher than the saline cohort, were not significantly different (and transgenic cDNA was not detected). This contrasts with the high dose cohort in which both viral genomes and derived cDNA were significantly different from the saline group at the end of the study.*

2. With the assumption that an autoreactive T cell population was generated as expected (see above concern), it is not clear that the beneficial effect of the gene therapy is local to the eye. The low dose treatment showed the same benefit as high dose treatment, but without similar evidence of local gene expression. An alternative hypothesis is that increased systemic IL-10 expression (in eye draining local lymph nodes or the spleen for example) could be responsible for the beneficial treatment effect. This could be addressed in the discussion and further mechanistic studies. Additional experiments to address this concern would be beyond the scope of this proof-of-concept study.

*Response: Also excellent comments and we added discussion points. Our hypothesis is that there is a limit to effective transduction of ocular tissue and the low dose AAV-IL10 was sufficient to locally inhibit the inflammatory response. In this case, perhaps more AAV vectors are not better; which is an increasingly observed phenomenon in animal models and in human patients following intravitreal administration of AAV vectors (PMIDs: 31544533, 30730541) .*

Statistical question:
Was clinical score data (Figure 1) analyzed per eye or per animal? Please clarify. If per eye, there needs to be an adjustment to account for non-independence of eye level data, or score could be assigned per animal by averaging both eyes.

*Response: Thank you. We clarified the data analysis to indicate that scores were assigned to each individual rat, not eye, by providing an average score per animal. This eliminated the between-eye correlation. See changes in statistical analysis section and figure legends.*

Comment:
Cell mediated killing of transduced cells is a concern for the safety and efficacy of gene therapy approaches. I was stuck by the appearance of the ciliary body in the high dose treatment group. There looks to have been considerable loss of CB tissue and thinning of the iris. Was this seen in multiple eyes, or is just this section abnormal?

*Response: We have re-done this histology figure with better representative images and labels. The changes observed in this section were interpreted to be processing and sectioning artifacts and not therapy related. A more representative image was provided.*

**Reviewer #2:** This manuscript reports intravitreal administration of an IL-10-AAV8 vector 7 days prior to inducing EAU in Lewis Rats, and the authors were able to detect IL-10 transcripts in ocular tissues and found a reduction in EAU and in the inflammatory cell infiltration in the IL-10-AAV8-treated EAU rats relative to BSS-injected EAU controls. They conclude this to be an effective therapy which should be considered for treating equine recurrent uveitis.

Comments:
1. How many experimental replicates were performed in this study? It would appear from the text that only one experiment was carried out with 14 rats divided into three treatment groups. Due to the variability in EAU in rodent models, it is expected that any treatment effect be reproduced at least 3 times. Can the authors please clarify in the Results how many repeat experiments were carried out.

*Response: The rat EAU model is well established in our laboratory and results from these experiments are reasonably consistent (see PMID:* ***31882729****). Based on these previous studies, we powered our study design to account for model induction variation.*

2. The use of the rat model in interesting as it is generally an acute form of ocular inflammation which eventually burns out over the course of a few weeks. Did the authors consider using a more recurrent form of EAU in mice? This might potentially be a more representative model with which to test this novel therapy.

*Response: Yes, we did consider other models such as recurrent EAU models in mice. However, for initial proof of concept studies, we elected to use a larger more consistent animal model to evaluate response to intravitreal AAV therapy.*

3. Given that rat EAU is a very acute, and clinically severe form of uveitis, it is nevertheless impressive that a suppression of EAU was observed using both doses of vector. Did the authors consider administering the treatment 7 days AFTER inducing EAU? This is a commonly used approach to determine whether a treatment can down regulate an already established disease, and should be discussed in respect of the current study.

*Response: Yes, we did consider a treatment arm instead of a preventative arm. However, because it generally takes 3-7 days to detect transgene expression after intravitreal injection of AAV vectors, we felt that we may miss the therapeutic effect with the short duration of the EAU in this model. In the next phases of this study, use of the viral vector in a more chronic model after development of uveitis is planned. We added discussion points on this subject in the discussion.*

4. The histology images in Fig 3 are too faint in my version to easily detect the infiltrating cells, and should be taken at a higher resolution. The retinal layers appear relatively unaffected, but have become detached from the choroid. Is this an artefact of the fixation procedure, or an effect of the EAU?

*Response: We have re-done this histology figure with better representative images and labels. The retinal detachment were interpreted to be processing and sectioning artifacts and not therapy or EAU related.*

5. Whilst I agree that it is good to detect IL-10 in the ciliary body and iris, the blood-retinal barrier at the level of the retinal endothelial cells is also a key part of the barrier. It would be helpful if semi-quantitative data for the IL-10 PCR in each tissue was shown. Data in Fig 4 is difficult to understand, as there is no reference to IL-10, only vector cDNA. Whilst I appreciate that the safety issues were of huge concern, it is also important to know whether, at the larger concentration, IL-10 could in fact be detected within the retinal tissues.

*Response: We apologize for the confusion – For these experiments, both RNA and DNA were isolated from the described ocular tissues. RNA was treated with DNAse and a first-strand cDNA synthesis kit was used to generate cDNA for each sample. Primers and a probe specific to the Equine-IL-10 sequence were used to amplify Equine-IL-10 transcripts using qPCR. Figure 4a depicts these results detecting transcripts specific to the equine form of IL-10 (i.e. vector cDNA in this case refers to the quantification of the equine IL-10 transcript isolated from each ocular compartment). The data presented in Figure 4b represents the qPCR results of the DNA isolated from each ocular tissue (both genomic DNA and vector DNA) using the same Equine-IL-10 specific primer/probe set and represents the biodistribution of the AAV-Equine-IL-10 vector.*

*Thus, as shown in Fig. 4a, the Equine-IL-10 transcript was detected in the retinal tissue at the high dose. We have amended the text in the results section and modified the figure legends to enhance the clarity of these results.*

While revising your submission, please upload your figure files to the Preflight Analysis and Conversion Engine (PACE) digital diagnostic tool, <https://pacev2.apexcovantage.com/>. PACE helps ensure that figures meet PLOS requirements.

*Response: We uploaded and formatted our figure files to the PACE digital diagnostic tool. The submitted figures were those generated by PACE.*
